# Influence of Probiotic Consortium on TH1 and TH2 Immune Response

**DOI:** 10.5195/cajgh.2013.122

**Published:** 2014-03-27

**Authors:** Gulnara Shakhabayeva, Almagul Kushugulova, Saule Saduakhasova, Samat Kozhakhmetov, Zhanagul Khasenbekova, Indira Tynybayeva, Talgat Nurgozhin, Zhaxybay Zhumadilov

**Affiliations:** Center for Life Sciences, Nazarbayev University, Astana, Kazakhstan

**Keywords:** probiotics, immune response, immunity

## Abstract

**Introduction:**

The main role of probiotics is to maintain homeostasis in the intestines and improve bowel protective function. The aim of the present study is to investigate immuno-modulatory effects of a probiotic consortium.

**Methods:**

Observations were carried out *in vitro*. The presence of IL-2, IL-4, IL-6, IL-8, IL-10, TNF-α, IFN-γ, IgA, IgM, and IgE was studied using a solid-phase enzyme immunosorbent assay on the VECTOR-BEST sets (Russia).

**Results:**

Immunomodulatory properties of the probiotic consortium were studied, which consisted of the following strains: *Streptococcus thermophilus, Lactococcus lactis, Lactobacillus plantarum, Lactobacillus fermentum, Lactobacillus acidophilus, Bifidobacterium longum, and Bifidobacterium bifidum.* Elevated concentrations of INFγ in control samples decreased 3.9 times (p < 0.05) after a saturation of blood with the probiotic consortium. Significant reduction of cytokine levels after the probiotic effects of the consortium was observed in IL-10 by 2.1 times (p < 0.05) and IgA by 1.87 times (p < 0.0005). There was a significant increase in the levels of IL-4, IgE, IL-6, and IL-8 by 1.3 (p < 0.005), 1.1 (p < 0.5), 18.0 (p < 0.005), and 6 (p < 0.05) times, respectively, in comparison with the control samples. IL-4 and INFγ have different effects on the synthesis of IgE. Soluble low affinity receptors FcɛRII (CD23) in association with IL-4 facilitate a differentiation of the B-lymphocytes in IgE-synthesizing cells, while γ-INF inhibits this process. It is known that the intracellular expression of γ-INF and IL-4 is the most reliable marker for Th1 and Th2 immune responses, respectively. The conducted studies determined that the ratio of INF-γ/IL-4 was 0.9 (control 4.8, P < 0.005) after the saturation of the blood cells with probiotic consortium. NF-γ/IL4 ratio decreased by 5.3 times compared with a control value, which indicates a reduction in the functional activity of Th1 type lymphocytes in comparison with the function of Th2 cells.

**Conclusion:**

The application of the probiotic consortium results in the maintenance of homeostasis by the stimulation of immune function through the activation of humoral immunity. Moreover, the probiotic application changes the orientation of the immunological memory causing the cancellation of the recruitment of Th1 cells in the response.

